# Effects of vitamin D supplementation on muscle strength in middle-aged and elderly individuals: a retrospective, propensity score-matched study

**DOI:** 10.3389/fnut.2024.1450265

**Published:** 2024-08-23

**Authors:** Peiyao Qi, Xiaomin Fu, Dan Zhao, Chunlin Li, Yanhui Lu, Nan Li

**Affiliations:** ^1^Department of Endocrinology, The Second Medical Center and National Clinical Research Center for Geriatric Diseases, Chinese PLA General Hospital, Beijing, China; ^2^Clinics of Cadre, Department of Outpatient, The First Medical Center, Chinese PLA General Hospital, Beijing, China; ^3^Department of Hematology, The Second Medical Center, Chinese PLA General Hospital, Beijing, China

**Keywords:** vitamin D, supplementation, muscle strength, retrospective studies, propensity score

## Abstract

**Introduction:**

The aim of this study is to investigate the impact of vitamin D supplementation on the muscle strength of the elderly.

**Methods:**

This retrospective, propensity score-matched study included 160 middle-aged and elderly individuals from a community in Beijing, China. The control group (n=110) received health education and lifestyle guidance, while the intervention group (n=50) was given oral vitamin D supplementation in addition to health education and lifestyle guidance. All participants underwent laboratory tests, muscle function, and physical function at baseline and follow-up.

**Results:**

In the propensity score-matched cohort of 41 patients per group, the levels of serum calcium and 25-hydroxyvitamin D in both groups were improved significantly by the end of the study (*p*<0.05), with the intervention group showing a more significant improvement. The muscle strength of the left lower limb in the intervention group significantly increased after the intervention (*p*<0.05). The results also showed that the grip strength and pinch strength of the patients in both groups increased after the intervention, and the difference between the two groups was statistically significant (*p*<0.05).

**Discussion:**

The findings of this study suggest that vitamin D supplementation, in conjunction with lifestyle guidance and health education, is beneficial for enhancing the upper and lower limb strength of patients.

## 1 Introduction

Vitamin D is a fat-soluble vitamin with multiple physiological functions, and its most critical role is promoting the absorption of calcium, magnesium, and phosphorus for normal skeletal development and maintenance (1). Vitamin D is synthesized in the skin, primarily through exposure to sunlight radiation. However, humans can also obtain a small amount of vitamin D through dietary intake or exogenous nutritional supplementation. Insufficient or deficient levels of vitamin D have been positively associated with various health risks, including myopathy, osteoporosis, as well as conditions such as rickets in adolescents and osteomalacia in adults (2). Numerous cross-sectional studies have reported that individuals with vitamin D deficiency commonly experience muscle pain, reduced muscle strength, decreased physical performance, an increased risk of falls, and alterations in muscle morphology (3–5).

Epidemiological investigations have revealed that a low vitamin D status is emerging as a very common condition in the global range, with the prevalence of vitamin D deficiency or insufficiency ranging from 30 to 60%, affecting over 1 billion individuals (6–8). Researchers found that globally, 15.7, 47.9, and 76.6% of people had serum 25-hydroxyvitamin D levels below 30, 50, and 75 nmol/L, respectively (9). Vitamin D is recognized as a pivotal nutritional factor in bone formation, garnering extensive attention for its integral role in bone metabolism. Insufficient vitamin D levels during adolescence may lead to delayed or aberrant skeletal development, and in severe cases, result in the occurrence of rickets. In middle-aged and elderly populations, vitamin D deficiency can contribute to impaired bone mineralization and osteoporosis, significantly elevating the risk of fractures and falls (10, 11). Moreover, due to hormonal fluctuations and alterations in lifestyle habits that accompany the aging process, a considerable number of middle-aged and elderly individuals present with abnormally low levels of serum 25-hydroxyvitamin D [25(OH)D] concentrations. Consequently, the issue of vitamin D deficiency in this particular demographic necessitates heightened scrutiny and consideration (12).

Bislev et al. conducted a systematic review and meta-analysis on the supplementation of vitamin D and its impact on muscle health. The results indicated that the current evidence does not support the beneficial effects of vitamin D supplementation on muscle health and may even suggest potential adverse effects (13). Increased muscle strength is regarded as a key factor in the prevention of falls and fractures, and improving the status of vitamin D may represent a straightforward approach to achieving these critical objectives (14). However, further clinical data is still required to establish the correlation between vitamin D supplementation and muscle strength conclusively.

Several randomized controlled trials (RCT) studies have been conducted to investigate the effect of oral vitamin D supplementation in older patients to prevent or treat sarcopenia, but the results are still controversial. Previously, a random control trial was conducted by the same team of this study to investigate the effects of stratified vitamin D supplementation in middle-aged and elderly individuals with vitamin D insufficiency in Beijing (15). It concluded that stratified vitamin D supplementation effectively increased the blood 25(OH)D level, as well as the number of cases with corrected vitamin D insufficiency or deficiency. In the RCT study, 247 observation variables have been collected and recorded in a research and development (R&D) database. Owing to the strict study design and case follow-up, the quality of this database is high and its data is more reliable than other databases such as electronic medical records (EMRs). Except the previous results, a number of other research directions could be explored by utilizing the same database of the RCT. This would save cost and time of the related research, and provide more evidence of vitamin D’s effectiveness and safety.

Based on real-world data with propensity score matching (PSM), we explored the relationship between vitamin D supplementation and muscle strength.

## 2 Methods

### 2.1 Study design

A total of 160 middle-aged and elderly people from a community in Beijing, China surveyed between March to April 2014 were retrospectively reviewed. Inclusion criteria comprised as follows: (a) According to the international universal assessment system for vitamin D status (16), the serum 25(OH)D level was used as an indicator. Vitamin D insufficiency was defined as 25(OH)D < 30 ng/mL. All participants had serum 25(OH)D levels below 30 ng/mL. (b) Participants were long-term residents of Beijing and aged 40–69 years old. Exclusion criteria: (a) hyperparathyroidism, chronic hepatic and renal insufficiency, malignant tumor; (b) use of medications (e.g., glucocorticoids) within 6 months; (c) use of vitamin D supplements, calcium supplements, thyrocalcitonin, bisphosphonates, anticonvulsants, anticoagulants, estrogen and estrogen receptor modulators that affect bone metabolism within 3 months; (d) poor glycemic control in diabetic patients; (e) Women preparing for pregnancy, pregnant women and lactating women. All included subjects voluntarily participated in this study and provided informed consent. Throughout the study, participants were given the option to withdraw from the study, and those who experienced serious adverse events during vitamin D supplementation were asked to discontinue participation in the study.

### 2.2 Grouping and intervention measures

A total of 160 subjects were included and divided into control group and intervention group. The control group (*n* = 110) received health education and lifestyle guidance. The intervention group (*n* = 50) received oral vitamin D supplementation in addition to health education and lifestyle guidance. Oral vitamin D3 supplements were provided by General Nutrition Corporation (GNC). The dose was stratified as follows: for vitamin D insufficiency [20 ng/mL ≤ 25(OH)D < 30 ng/ ml], oral vitamin D3 supplement was given at 5,000 IU/w; for mild vitamin D deficiency [12 ng/mL ≤ 25(OH)D < 20 ng/mL], oral vitamin D3 supplement was given at 10,000 IU/w; for severe vitamin D deficiency [25(OH)D < 12 ng/mL], oral vitamin D3 supplement was given at 15,000 IU/w. The Ethics Committee of Chinese PLA General Hospital approved this study.

### 2.3 Data collection

A questionnaire survey was conducted on all participants by specially trained staff to collect baseline information, encompassing general conditions, lifestyle, past medical history, and medication history. During the research, a follow-up was conducted every 2 weeks on all participants by the trained staff and adverse events were inquired. The adverse events following oral vitamin D administration were defined as toxicity symptoms caused by an overdose of vitamin D, with early manifestations including nausea, bone and joint pain, skin-itching, hypercalcemia, and abnormalities in kidney and liver function. All biochemical indicators were reassessed for safety evaluation 3 months after the intervention. The intervention period lasted for a total of 9 months, ending from March to April 2015. To avoid the influence of seasonal fluctuations of 25(OH)D on the test results, the determination of the terminal level of biochemical indicators was conducted in the same month as the baseline level.

### 2.4 Physical examination

Body height and weight (exactly 0.5 cm and 0.5 kg, respectively) were measured uniformly and strictly by trained staff using calibrated and qualified equipment. The participants were requested to be in a fasted state, remove their shoes, and wear light clothing. BMI was calculated as follows: weight (kg)/ height (m^2^). According to the Seventh Report of the Joint National Committee (JNC), systolic and diastolic blood pressure were measured. The upper right brachial arterial pressure was measured once every 2 min with a mercury column sphygmomanometer, and the average value was recorded for 3 times in total.

### 2.5 Sample collection and laboratory tests

Fasting venous blood samples were collected at 6:00–8:00 h the next day after an 8–10 h fast. Biochemical measurements were completed within 1 week after sampling. Roche automatic biochemical analyzer (Cobas E601, Roche) was used to detect the indicators including calcium (Ca), phosphorus (P), high-density lipoprotein cholesterol (HDL-C), low-density lipoprotein cholesterol (LDL-C), etc. Serum 25 (OH) D levels were measured by electrochemiluminescence (Roche Cobas C6000, Roch Diagnostics GmbH).

### 2.6 Muscle strength and physical function measures

Grip strength of the participants’ handedness was measured using the Jamar grip device (Sammons Preston, United States). The participants were explained how to use the handgrip device, and the formal measurement was started after one exercise. Pinch strength of the participants’ handedness was measured using the Jamar pinch device (Sammons Preston, United States). During the process, the participants used the thumb and index finger to press the pinch device relative to each other in a sit-to-stand position, and continued to hold the device with maximum force for a few seconds before release. Rest for at least 3 min between each test to avoid muscle fatigue. Both the grip and pinch strength of the dominant hand was measured three times in a row, and the average of the three measurement results was taken in Kilogram (Kg).

The lower limb muscle strength of the participants was measured using a portable hand-held muscle strength dynamometer (Hoggan Health Industries, Inc., United States). The quadriceps muscle strength was used to represent the lower limb muscle strength. Participants were seated with a chair height of 46 cm and joint and knee flexion of 90°. The dynamometer was placed on the lower leg near the ankle joint to measure bilateral lower limb muscle strength. Each side was measured three times, and the average of the three measurements was taken in Newton (N).

The timed up and go test (TUG) and timed chair stands (TCS) were used to assess lower physical function (16). TUG measures the amount of time it takes to get up from a chair, walk 3 m, and return to a sitting position without any physical help. Two trials of the TUG were performed by each participant. The TCS test can be used as a simple alternative to measure the strength of lower limbs, mainly to measure the strength of quadriceps femoris. A seat with a height of about 46 cm was used for measurement. Participants were seated with their arms folded across their chests and asked to complete 5 sit-to-stand movements without stopping or using their arms as fast as possible. Increasing time reflect worse performance and a decline in function.

### 2.7 Statistical analysis

Data collection was blinded. Data collected by two trained researchers using Epidata software for entry. Statistical analysis was performed using the software package SPSS Statistics version 26.0 (IBM Corp., Armonk, N.Y., United States), with a significance level set at *p* < 0.05. Quantitative characteristics were expressed as mean ± standard deviation (SD), and intergroup comparison was performed using. The Kolmogorov–Smirnov test was applied to test normal distribution. Non-parametric Wilcoxon test was used for data that did not conform to a normal distribution. A *p*-value < 0.05 indicated significant difference.

This study was retrospective and data collection was not strictly randomized, which inevitably led to confounding factors. It was not possible to determine directly whether the changes in muscle strength among the participants were caused by vitamin D supplementation. Therefore, to ensure comparability between the experimental and control groups and to minimize the influence of confounding factors, the two groups were propensity score matched in terms of sex, drinking history, body mass index (BMI), and other parameters. We used propensity score-matching to compare selected baseline variables with variables with similar characteristics and expected risk in the two groups.

## 3 Results

An overview of the baseline characteristics of the study population before and after the propensity score-matching is presented in Table 1. Among the 160 middle-aged and elderly people included, 50 (31.25%) received vitamin D supplementation, and the remaining 110 (68.78%) were in the control group before the propensity score matching. There were significant differences in gender, waist-hip ratio (WHR) and diastolic blood pressure (DBP) between the two groups (*p* < 0.05). With the group as the dependent variable and gender, age, smoking history, drinking history, hypertension, glucose metabolism, BMI, and WHR as the independent variables, the nearest neighbor matching method was used to conduct a 1:1 propensity score-matching. Logistic regression was used to calculate the propensity score, and the matching tolerance was 0.02. After propensity score-matching, 41 pairs of fuzzy matching cases were found with no exact matching cases. A total of 41 pairs were successfully matched, and 9 cases failed to find an effective matching population. There was no significant difference in general data between the two groups (*p* < 0.05).

**Table 1:** Baseline characteristics of the study groups.

Parameter	Before PS matching (*N* = 160)			After PS matching (*N* = 82)		
	Control group (*N* = 110)	Intervention group (*N* = 50)	*p*-value	Control group (*N* = 41)	Intervention group (*N* = 41)	*p*-value
Age, years	49.75 ± 4.92	49.26 ± 7.30	0.670	49.98 ± 4.97	49.76 ± 7.75	0.879
Males, % sex (f/m)	95 (86.36)	31 (62.00)	**<0.001**	31 (75.61)	31 (75.61)	1.000
Smoker, %	39 (35.45)	11 (22.00)	0.089	8 (19.51)	11 (26.83)	0.432
Alcohol, %	74 (67.27)	33 (66.00)	0.874	29 (70.73)	28 (68.29)	0.810
Coffee, %	25 (22.73)	12 (24.00)	0.860	12 (29.27)	8 (19.51)	0.304
Tea, %	51 (46.36)	25 (50.00)	0.669	17 (41.46)	21 (51.22)	0.376
Hypertension, %	22 (20.00)	8 (16.00)	0.548	8 (19.51)	7 (17.07)	0.775
Diabetes mellitus, %	11 (10.00)	3 (6.00)	0.407	6 (14.63)	3 (7.32)	0.289
Body mass index, kg/m^2^	25.52 ± 3.03	24.51 ± 3.53	0.066	25.47 ± 2.85	24.64 ± 3.28	0.225
Waist hip rate	0.88 ± 0.06	0.84 ± 0.08	**0.011**	0.86 ± 0.06	0.86 ± 0.07	0.719
SBP, mmHg	132.35 ± 14.82	127.53 ± 17.84	0.076	132.60 ± 15.18	127.75 ± 17.81	0.189
DBP, mmHg	83.02 ± 9.62	78.66 ± 9.82	**0.009**	81.57 ± 10.32	79.73 ± 9.98	0.415

SBP, systolic blood pressure; DBP, diastolic blood pressure.

Bold values (when *p* is less than 0.05) means it is statistically significant.

After propensity score matching, the comparison of serum 25(OH)D levels between the two groups showed a statistically significant difference (*p* < 0.05), while other indicators did not show significant differences in baseline data (Table 1). The BMI, waist-to-hip ratio, biochemical indicators, muscle strength, and physical function of the participants in both groups were compared before and after the intervention. The results showed that at the end of the study, the levels of serum calcium and 25(OH)D in both groups were improved (*p* < 0.05), with the intervention group showing a more significant improvement (Table 2). The muscle strength of the left lower limb in the intervention group significantly increased after the intervention (*p* < 0.05). Although there were significant differences in the TUG and TCS test results of the patients in both groups before and after the intervention, there was no improvement reflected. The study also showed that the grip strength and pinch strength of the patients in both groups increased after the intervention, and the difference between the two groups was statistically significant (*p* < 0.05) (Table 3).

**Table 2:** Changes of laboratory values after vitamin D supplementation.

Parameter	Control group (*N* = 41)				Intervention group (*N* = 41)				*p*-value^*^
	Baseline	At the end of research	Difference	*p*-value	Baseline	At the end of research	Difference	*p*-value	
Ca, mmol/L	2.33 ± 0.07	2.38 ± 0.10	0.05 ± 0.13	**0.012**	2.33 ± 0.08	2.39 ± 0.08	0.06 ± 0.11	**0.002**	0.778
P, mmol/L	1.10 ± 0.13	1.10 ± 0.15	0.00 ± 0.19	0.893	1.07 ± 0.14	1.14 ± 0.14	0.07 ± 0.22	0.050	0.106
Serum 25(OH)D, (ng/ml)	18.65 ± 6.10	22.76 ± 11.20	4.11 ± 12.58	**0.043**	14.39 ± 4.23	21.49 ± 9.45	7.10 ± 10.61	**<0.001**	0.249
PTH, pg./ml	41.80 ± 13.27	36.37 ± 12.11	−5.43 ± 19.13	0.076	39.74 ± 13.01	40.59 ± 15.74	0.85 ± 20.28	0.789	0.153
ALP, U/L	59.91 ± 19.58	68.48 ± 14.86	8.57 ± 23.48	**0.024**	66.62 ± 13.95	73.20 ± 19.03	6.58 ± 23.28	0.078	0.701
HDL-C, mmol/L	1.47 ± 0.40	1.45 ± 0.46	−0.02 ± 0.58	0.865	1.41 ± 0.33	1.32 ± 0.34	−0.08 ± 0.44	0.228	0.550
LDL-C, mmol/L	3.36 ± 0.87	3.36 ± 0.76	0.00 ± 1.33	1.000	3.12 ± 0.78	3.50 ± 0.84	0.38 ± 1.14	**0.037**	0.164

Ca, calcium; P, phosphorus; 25(OH)D, 25-hydroxyvitamin D; HDL-C, high-density lipoprotein cholesterol; LDL-C, low-density lipoprotein cholesterol.

Values are presented as mean ± standard deviation. All values were normally distributed. Bold values (when *p* is less than 0.05) means it is statistically significant.

*p represents the statistical difference in the comparison of inter-group differences.

**Table 3:** Changes of muscle function after vitamin D supplementation.

Parameter	Control group (*N* = 41)				Intervention group (*N* = 41)				*p*-value^*^
	Baseline	At the end of research	Difference	*p*-value	Baseline	At the end of research	Difference	*p*-value	
Body mass index, kg/m^2^	25.47 ± 2.85	24.77 ± 3.36	−0.70 ± 3.95	0.265	24.64 ± 3.28	25.69 ± 3.33	1.05 ± 5.06	0.193	0.086
Waist hip rate	0.86 ± 0.06	0.87 ± 0.07	0.01 ± 0.07	0.574	0.86 ± 0.07	0.88 ± 0.06	0.02 ± 0.10	0.158	0.427
Grip strength, kg	33.83 ± 11.05	30.91 ± 10.71	−2.92 ± 13.70	0.181	30.41 ± 9.90	34.03 ± 8.82	3.63 ± 11.82	0.056	**0.023**
Pinch strength, kg	24.70 ± 4.62	21.72 ± 5.92	−2.98 ± 7.64	**0.017**	23.15 ± 4.62	23.47 ± 4.51	0.32 ± 5.68	0.722	**0.030**
Lower limb (left), *N*	39.50 ± 8.27	41.20 ± 8.54	1.70 ± 12.14	0.376	38.75 ± 8.16	42.78 ± 6.71	4.03 ± 9.85	**0.012**	0.342
Lower limb (right), *N*	40.55 ± 10.07	39.10 ± 11.12	−1.45 ± 15.12	0.543	40.44 ± 8.31	43.94 ± 11.46	3.50 ± 15.00	0.143	0.140
Timed Up &Go^a^, sec	7.71 ± 1.18	7.05 ± 0.90	−0.66 ± 1.59	**0.011**	8.06 ± 1.10	6.77 ± 0.92	−1.29 ± 1.68	**<0.001**	0.087
Timed Chair Stand^b^, sec	7.49 ± 1.23	6.92 ± 1.25	−0.57 ± 1.51	**0.020**	7.83 ± 1.51	6.63 ± 1.25	−1.20 ± 1.94	**<0.001**	0.104

a Timed Up & Go is the amount of time (seconds) it takes to rise from a chair, walk 3 m, and return to a sitting position.

b Timed Chair Stands is the time (seconds) it takes to complete 5 chair stands without stopping or using arms to push off. Increased time to perform these tests reflected poorer physical function.

Values are presented as mean ± standard deviation. All values were normally distributed. Bold values (when *p* is less than 0.05) means it is statistically significant.

*p represents the statistical difference in the comparison of inter-group differences.

## 4 Discussion

In recent years, increasing evidence has shown that vitamin D status can modulate musculoskeletal effects on muscle health, especially on muscle mass and muscle function in the elderly with vitamin D deficiency and in patients with chronic diseases (17, 18). However, the physical effects of vitamin D supplementation remain unclear because many factors may affect the improvement in physical function, including physical performance level, physical activity level, race, vitamin D status, and type of exercise. Currently, there is still a lack of clear consensus on the association between vitamin D status and muscle mass or strength. Previous studies have yielded conflicting conclusions about whether vitamin D supplementation improves muscle strength (19). In 2017, Iolascon et al. conducted a study of 113 postmenopausal women with osteoporosis or vitamin D deficiency and found that 6 months of active vitamin D supplementation significantly improved muscle strength in their upper and lower limbs and reduced the incidence of falls (20). However, a randomized, double-blind, placebo-controlled study showed no benefit of vitamin D supplementation for muscle strength in postmenopausal women (21). In a randomized, double-blind, placebo-controlled trial in a deficient dialysis population, vitamin D3 supplementation had no effect on muscle strength (22). Aschauer et al. found that vitamin D3 supplementation had no effect on regulating or improving muscle strength endurance, functional ability, or aerobic capacity (23). In our study, although there was no statistically significant difference in the change of upper limb strength between the control and intervention groups, both groups showed an increase in grip and pinch strength after the intervention, with the difference between the two groups being statistically significant. Moreover, the intervention group exhibited a significant improvement in the strength of the left lower limb (*p* < 0.05), indicating that vitamin D3 supplementation is beneficial for the maintenance and enhancement of both upper and lower limb strength.

Handgrip strength tests are widely used as a measure of muscle strength and have become an outcome measure of nutritional status. However, in previous studies, the results were inconsistent regarding the role of vitamin D in grip strength. Studies have shown that malnutrition and vitamin D deficiency are associated with weaker grip strength. In this study, the upper limb muscle strength of participants was assessed by measuring grip strength (24–26). This study found that after propensity score matching, vitamin D3 supplementation improved upper grip strength, as well as hand muscle pinch strength in middle-aged and elderly participants. In this study, the baseline serum 25(OH)D levels in the intervention group were lower than those in the control group. Furthermore, at the conclusion of the follow-up period, the difference in the change of 25(OH)D levels in the intervention group was greater than that of the control group. At the end of the follow-up, there was a significant increase in serum 25(OH)D concentrations in both groups (*p* < 0.05), with the intervention group showing a greater increase in serum 25(OH)D concentration than the control group. This is consistent with the results of muscle strength measurements. However, the TUG and TCS test results showed no improvement, which we speculate may be related to factors such as the age of the subjects, compliance, baseline 25(OH)D levels, body absorption, and lifestyle. However, the specific reasons and correlations require further investigation. The muscle strength measurement results in this study indicate that vitamin D supplementation can enhance upper limb strength. We have reported this phenomenon in our study and plan to delve deeper into the mechanism in future research.

Oral active vitamin D (calcifediol) has a higher intestinal absorption rate than regular vitamins, which may have important advantages in situations where intestinal absorption capacity is reduced due to various diseases. Cholecalciferol or vitamin D3 is the most commonly used form of supplement today. Relevant literature reports that the cost of active vitamin D treatment is about 3.6 times that of regular vitamin treatment (27). Although the treatment cost of active vitamin D is significantly higher than that of ordinary vitamin D, a meta-analysis study showed that there was no significant difference in efficacy and safety between the two groups in studies with the number of new vertebral and non-vertebral fractures as the primary outcome (28). For muscle strength comparison, in a double-blind RCT study, active vitamin supplementation was 2.8 times more effective than vitamin D3 supplementation in improving lower-extremity muscle strength in healthy white postmenopausal women (29).

Feng et al. (30) evaluated the effects of regular vitamin D and active vitamin D interventions on physical function, muscle strength, and fall risk in elderly patients with osteoporosis. The results show that active vitamin D can improve the physical ability of patients, reduce the risk of falls, and effectively improve the bone mass of the lumbar spine in patients with osteoporosis, while the improvement of the bone mass of the lumbar spine and hip of patients with ordinary vitamin D is not obvious. The results of the study found that the effect of vitamin D supplementation on muscle strength was relatively improved after 6 and 12 months of intervention, but there was no significant difference between the effects of the two vitamin D supplements.

Vitamin D deficiency has been shown to impair muscle function, but the association of vitamin D deficiency with cardiopulmonary endurance is uncertain. A 19-week controlled intervention study in India found that vitamin D3 supplementation did not improve muscle strength and cardiopulmonary endurance in healthy young Indian men with vitamin D deficiency (31). A meta-analysis published in 2017 only included RCT studies that used grip strength and TUAG as indicators of muscle function (15 RCTS, 2408 people) and did not find an improvement effect of vitamin D3 supplementation, with significant heterogeneity among studies (32).

Vitamin D supplementation plays an important role in the treatment of osteoporosis. Although the guidelines include vitamin D supplementation as a recommendation, there is still a lack of high-quality evidence to confirm the difference and priority of choice between active vitamin D and regular vitamin D. In 2019, Xiao et al. (28) conducted a meta-analysis to explore the difference between regular vitamin D and active vitamin D in reducing the risk of fractures in patients with osteoporosis, and no difference was found between the two. Calcifediol supplementation is more effective and faster at increasing 25 (OH) D serum levels than cholecalciferol and is associated with greater improvements in muscle function.

In terms of statistical methods, this study is retrospective, and potential limitations of the database in terms of data completeness, and limitations in the availability of historical medical data are unavoidable. In addition, due to the non-intervention study design and the inherent limitations of observational research, the results of this study are susceptible to bias, such as selection bias and limited access to historical medical data. After propensity score matching, there was no difference in demographic data between the control group and the intervention group, which excluded confounding factors to a certain extent.

In this study, residual confounding cannot be ruled out, given that the treatments in the two groups will be assigned without randomization. Although this study will use a propensity score approach to derive a comparison group that is more balanced in terms of measured baseline characteristics and known confounders, achieving comparability and eliminating possible selection bias, residual confounding is still possible. Due to the limited number of patients and the retrospective nature of the data collection, the results should be interpreted with caution. However, further studies with larger sample size and longer follow-up time are needed to confirm the results.

## 5 Conclusion

In this study, we found that vitamin D3 supplementation was associated with improvement of upper and lower limb strength, delayed muscle strength decline, and was beneficial for maintaining muscle strength. These findings provide evidence that warrants confirmation in future intervention studies with a larger sample size and longer follow-up.

## Data Availability

The raw data supporting the conclusions of this article will be made available by the authors, without undue reservation.
